# Randomized Clinical Trial investigating Self-Assembling Peptide P_11_-4 for Treatment of Early Occlusal Caries

**DOI:** 10.1038/s41598-020-60815-8

**Published:** 2020-03-06

**Authors:** Dafina Doberdoli, Claudine Bommer, Agim Begzati, Fehim Haliti, Monika Heinzel-Gutenbrunner, Hrvoje Juric

**Affiliations:** 1 Department of Pediatric Dentistry and Prevention, University Dentistry Clinical Center of Kosovo, Rrethi I spitalit p.n, Pristina, 10000 Kosovo; 2 School of Dental Medicine - Department of Pediatric Dentistry and Prevntion, University of Zagreb, Gundulićeva 5, Zagreb, 10000 Croatia; 3 credentis ag, Dorfstrasse 69, 5210 Windisch, Switzerland; 4 MH Statistics, 35041 Marburg, Germany

**Keywords:** Minimal intervention dentistry, Randomized controlled trials

## Abstract

Non-invasive caries treatment is a major focus in modern dentistry. The present study was designed to evaluate the effectiveness of monomeric self-assembling peptide P_11_-4 (SAP P_11_-4) in combination with fluoride varnish or polymeric self-assembling peptide matrix (SAPM) in treatment of non-cavitated occlusal caries. Ninety children and adolescents were included in this randomized, gold-standard-controlled clinical trial. Test Group 1 received SAP P_11_-4 and twice fluoride varnish at baseline and Day 180, Test Group 2 received SAP P_11_-4 on baseline and twice weekly SAPM (home-application), and Control Group received fluoride varnish on baseline and Day 180. Caries progression was measured by laser fluorescence, Nyvad Caries Activity, ICDAS-II-codes, and investigator assessments. Laser fluorescence changes demonstrated superior results for Test Group 1 and 2, as values decreased compared to an increase for the Control Group (p < 0.0005). ICDAS-II codes at Day 360 showed partial regression for Test Group 1 (6.7%) and Test Group 2 (20.0%) and partial progression for Control Group (23.3%) (p < 0.01). Nyvad Caries Activity yielded superior caries inactivation for Test Groups, compared to Control Group (p = 0.002). This trial showed that SAP P_11_-4, applied either in combination with fluoride varnish or twice weekly SAPM, was a superior treatment for early caries compared to fluoride varnish alone.

## Introduction

Caries levels have significantly decreased over the past decades, partially due to the introduction of fluoride in various forms^1^. However, despite the promising efforts and results worldwide, caries levels have remained high, and caries is still the most common disease worldwide^2^. Recent efforts to lower caries levels have focused on treating early carious lesions non-invasively, as there is consensus that only early intervention, prior to cavitation, can lead to a regression of the caries to a more healthy state^3,4^. Novel therapeutics have been called for, and promising approaches are based on biomimetic research and development. Biomimetic concepts fall into two categories, either based on amelogenin or derivatives thereof, or on rationally designed and screened systems^5,6^. There is substantial research in the field, yet most of it is *in vitro*-based. The exceptions are recent clinical investigations of the rationally designed self-assembling peptide P_11_-4 applied either in its monomeric form (SAP P_11_-4)^5,6^ or its polymeric form as a self-assembled peptide matrix (SAPM). This is the first clinical trial investigating the combined effect of SAP P_11_-4 and SAPM in treatment of early carious lesions.

SAP P_11_-4 has been systematically investigated^7^. The self-assembling peptide was rationally designed to provide favourable physicochemical characteristics for nucleation of hydroxyapatite (HA) on the surface of the formed fibres of the 3D matrix^8,9^. The mechanism of action of SAP P_11_-4 in the remineralization of enamel was proposed early on and has been proven since^10,11^. When monomeric SAP P_11_-4 is applied onto the carious lesion surface, the SAP P_11_-4 peptide diffuses into the lesion and self-assembles within the lesion into a 3D matrix, followed by attraction of minerals from saliva to form new HA or fluoridated HA crystals^7^.

*In vitro* experiments on SAP P_11_-4 have demonstrated superior remineralization to saliva, fluoride varnish and casein phosphopeptide - amorphous calcium phosphate (CPP-ACP)^11–14^. Microhardness measurements as a function of the enamel lesion depth have shown that remineralization after SAP P_11_-4 application occurs deep into the lesion body. In contrast, mineralization with fluoride is restricted to the outer enamel layer^15^.

For SAP P_11_-4 to be translated into daily clinical practice, it is essential that clinicians can visualize the treatment effect with available diagnostics. Both *ex vivo* and *in vivo* studies have reported the successful use of laser fluorescence, PTR-LUM, Impedance, QLF and radiography in monitoring remineralization after SAP P_11_-4 application in a time-dependent manner^12,13,16–20^.

Yet, the ultimate test for any device is the clinical efficacy within daily clinical practice. Early clinical trials have proven the safety of SAP P_11_-4 and have indicated the efficacy of the product^19,21^. Recent reports on randomized controlled clinical trials demonstrated superior efficacy compared to fluoride varnish, placebo or saliva in the treatment of initial caries – both in combination with fluoride varnish or with normal oral hygiene, including fluoride toothpaste^16,18,22,23^.

Two clinical trials are of particular interest. The first investigated a combination of SAP P_11_-4 and fluoride varnish in remineralisation of early carious lesions, with results showing superior remineralisation, caries inactivation and regression of SAP P_11_-4 and fluoride varnish compared to fluoride varnish alone, yet raising several clinically relevant question^16^: Is there an additive, synergistic, or inhibiting effect between SAP P_11_-4 used alongside fluoride varnish? Could fluoride varnish be substituted with a home care solution avoiding risks and logistic difficulties associated with high fluoride application? Is the efficacy of SAP P_11_-4 and fluoride varnish in fully erupted molars and premolars different to that of erupting molars (as only erupting molars were treated)? Is the regenerative process supported by SAP P_11_-4 fully completed at the 6 month recall – the last recall of the study?

A second publication reported an *in situ* trial investigating the use of a gel including SAP P_11_-4 in its matrix form (SAPM) in the prevention of demineralisation and treatment of early demineralised lesions. The results suggested that SAPM gel with 900ppm fluoride and twice a week application showed equal or superior efficacy in high risk caries patients compared to fluoride varnish^24^.

P_11_-4 within the SAPM gel was developed for home treatment of caries and prevention thereof, primarily by inhibiting demineralisation, whereas the monomeric SAP P_11_-4 product for professional use was specifically developed for treatment of initial carious lesions. SAPM and SAP P_11_-4 differ in their primary mode of action, formulation and concentration of P_11_-4. Whereas SAP P_11_-4 diffuses into the lesion body upon application on the tooth surface of active carious lesions, the location of action of SAPM is primarily the tooth surface protecting the tooth minerals, as the fibres are too long to diffuse into the lesion body.

In the present clinical study, we investigated for the first time the possibility of substituting the use of high concentration fluoride (i.e. fluoride varnish) with an SAPM gel including toothpaste levels of fluoride (900 ppm Fluoride) suitable for home use. Furthermore, this is the first clinical study investigating the combinational of SAPM and SAP P_11_-4. We based the design on a previous clinical trial, which studied a combination of SAP P_11_-4 and fluoride varnish (SAP P_11_-4+Fluoride Varnish; Test Group 1) and compared it to fluoride varnish only (Fluoride Varnish; Control) in the treatment of children and adolescents. Yet, we extended the study design, using the same control group, and the previous test group 1 as a positive control, adding a third treatment group of SAP P_11_-4 and twice weekly home application of an SAPM (SAP P_11_-4+SAPM; Test Group 2) and a longer follow-up period, and a wider patient selection. We could thus address the clinically relevant questions raised by the previous trial: The efficacy of SAP P_11_-4 without simultaneous application of fluoride varnish. The efficacy of SAP P_11_-4 in treatment of initial occlusal caries of fully erupted molars and premolars, and determine the time in which the SAP P_11_-4 supported remineralisation is completed and whether it is stable over 12 months.

We tested the hypothesis that SAP P_11_-4+Fluoride Varnish and SAP P_11_-4+SAPM would show no difference in clinical success in the treatment of early occlusal caries and that both would be superior to the current clinical gold-standard of fluoride varnish alone – corroborating data from the previous clinical trial^16^ and providing an alternative for the clinician to substitute semi-annual high fluoride varnish application with a twice weekly application of an SAPM gel.

## Results

The present study was approved by the University Dentistry Clinical Center of Kosovo ethical committee on May 27, 2014 (Project Number: 500; Chairman: Prof. Ass. Dr Fatmir Dragidella) and was registered at clinicaltrials.gov (NCT03780270 – 19/12/2018). Patient recruitment started on June 16, 2014 (First patient’s first visit) and lasted until Dec 31, 2016 (Last patient’s last visit).

Ninety children and adolescents were recruited into the trial and equally assigned at random to the three study groups (Fig. S1). None of the baseline values showed statistical differences among the groups (Table 1). Throughout the study, 7 patients in the Control Group were lost. One patient was lost after baseline, two patients were lost due to clinically indicated placement of a filling at the study lesion after completion of the Day 90 visit, and four more were lost after the Day 180 visit. An additional 4 lesions in the Control Group required a filling after completion of the study visit at Day 360. For those patients, all assessments are available, including Day 360 assessments. Test Group 1 lost 3 patients throughout the study, one each after Baseline, Day 90 and Day 180. Test Group 2 lost 3 patients throughout the study; two patients were lost after Day 90, and one lesion required filling after Day 180 (Fig. S1).

**Table 1:** Baseline Characteristics.

	Control Group (30)	Test Group 1 (30)	Test Group 2 (30)
**Demographics**			
Age	11.6 ± 2.9	11.9 ± 2.2	12.0 ± 2.0
Sex (female)	17 (56.7%)	20 (66.7%)	21 (70%)
**Oral Characteristics**			
DMFT	2.7 ± 1.2	2.9 ± 1.4	3.7 ± 2.6
**Plaque Index**			
Plaque Index	1.38 ±0.58	1.30 ±0.40	1.41 ±0.62
**Oral Hygiene Index**			
Very good	2	0	1
Good	11	16	13
Fair	9	12	13
Poor	8	2	3
**Caries Risk**			
High	7 (23.3%)	4 (13.3%)	6 (20.0%)
Moderate	23 (76.7%)	26 (86.7%)	24 (80.0%)
**Tooth Specification**			
Premolar/Molar	3/27	9/21	4/26
Mandible/Maxilla	12/18	15/15	14/16
Fully erupted	29 (96.7%)	29 (96.7%)	28 (93.3%)
In eruption	1 (3.3%)	1 (3.3%)	2 (6.7%)
**ICDAS-II at Baseline**			
2	19 (63.3%)	19 (63.3%)	24 (80.0%)
3	11 (36.7%)	11 (36.7%)	6 (20.0%)
**Nyvad Activity Score at Baseline**			
Active (Score 1, 2, 3)	26 (86.7%)	29 (96.7%)	27 (90%)
Inactive (Score 4, 5)	4 (13.3%)	1 (3.3%)	3 (10.0%)
**VAS Texture**			
Mean (STD)	30.3 (±15.5)	36.1 (±7.6)	30.5 (±11.7)

### Laser fluorescence

Change in the laser fluorescence values was the primary parameter (Table 2). The laser fluorescence values at baseline were: Control Group, 27.2 ± 6.6; Test Group 1, 31.4 ± 6.5; Test Group 2, 25.3 ± 7.1. The difference in baseline values for Control Group vs. Test Group 1 respectively Test Group 1 vs. Test Group 2 was accounted for within the statistical analysis. In the follow-up visits, the Control Group’s laser fluorescence values increased by 5.5 ± 7.8 to 31.8 ± 6.4 on Day 360, decreased by −8.5 ± 5.9 to 22.7 ± 4.3 in Test Group 1 and by −7.7 ± 7.8 to 17.6 ± 5.1 in Test Group 2. Test Group 1 and Test Group 2 both showed superior results at every follow-up compared to the Control Group (p < 0.0005) but there were no statistical differences between Test Group 1 and Test Group 2 (p = 0.595).

**Table 2:** Laser Fluorescence Readings throughout the Study Duration given as Averaged Values from three Readings and as Changes to Day 0.

	Control Group		Test Group 1		Test Group 2		Test 1 vs Control Test 2 vs Control*
	Values	Change to Day 0	Values	Change to Day 0	Values	Change to Day 0	
**Day 0**							
Mean	27.2 ± 6.6		31.4 ± 6.5		25.3 ± 7.1		
Median	27.2		30.7		24.0		
95% CI	24.8–29.7		28.9–33.8		22.6–27.9		
**Day 90**							
Mean	30.2 ± 6.6	3.3 ± 6.0	26.9 ± 5.8	−4.4 ± 4.1	19.4 ± 6.4	−5.9 ± 4.7	**p < 0.0005**
Median	31.3	1.7	26.7	−4.0	19.7	−4.8	**p < 0.0005**
95% CI	27.7–32.7	1.0–5.5	24.7–29.1	(−6.0)–(−2.9)	17.0–21.8	(−7.6)–(−4.1)	
**Day 180**							
Mean	30.4 ± 5.7	4.2 ± 6.0	24.8 ± 4.7	−6.7 ± 4.8	18.8 ± 5.9	−6.6 ± 6.5	**p < 0.0005**
Median	31.0	3.7	24.5	−6.2	19.0	−7.3	**p < 0.0005**
95% CI	28.0–32.9	1.6–6.8	23.0–26.7	(−8.5)–(−4.8)	16.6–21.1	(−9.1)–(−4.1)	
**Day 360**							
Mean	31.8 ± 6.4	5.5 ± 7.8	22.7 ± 4.3	−8.5 ± 5.9	17.6 ± 5.1	−7.7 ± 7.8	**p < 0.0005**
Median	32.3	5.0	22.0	−7.7	18.7	−6.0	**p < 0.0005**
95% CI	29.0–34.6	2.2–8.9	21.0–24.4	(−10.8)–(−6.2)	15.6–19.6	(−10.8)–(−4.6)	

*Tested effect: Interaction in months and treatment group with hierarchical linear model (HLM); Test Group 1 vs Test Group 2 did not yield significant differences (p = 0.595).

Changes are tested in an ANCOVA-model; Adjusted for laser fluorescence value at Day 0, sex, age, plaque index at Day 0.

### ICDAS-II code and lesions requiring restorative intervention

All three study groups had distributions between ICDAS-II codes 2 and 3 (Table 1) at baseline. The changes in ICDAS-II Code of the study lesions are shown in Table 3. Throughout the study period, none of the control lesions regressed into a lower caries class; however, until Day 360, 9 (30%) lesions increased by at least one class (Day 360: 7 increases and 2 lesions that needed filling after Day 90). None of the Test Group 1 or 2 lesions moved into a higher ICDAS-II Code, including the lesion in Test Group 2, which was restored after the Day 180 visit upon request of the guardian. In Test Group 1, two lesions (6.7%) regressed into a smaller ICDAS-II code by Day 360; in Test Group 2, six lesions (20%) regressed into a smaller ICDAS-II code. There was no statistical significant difference between Test Group 1 & 2 (p = 0.26), but both Test Groups 1 & 2 performed superior to the control (p < 0.05). Throughout the study, 6 (20%) of the lesions in the Control Group required restorative intervention (2 after Day 90 and 4 after Day 360); no lesions were restored in Test Group 1, and one lesion was restored in Test Group 2 after Day 180.

**Table 3:** ICDAS-II Codes and Code Changes in Control and Test Groups at baseline and after 90, 180, and 360 days follow-up.

ICDAS-II Class and Class Change	Control Group		Test Group 1		Test Group 2	
	n(%)	Missing (%)/ Filled^b^(%)	n(%)	Missing (%)/ Filled^b^(%)	n(%)	Missing (%)/ Filled^b^(%)
**Baseline**	30(100)		30(100)		30(100)	
2	19(63.3)		19(63.3)		24(80)	
3	11(37.7)		11(37.7)		6(20)	
**Day 90**	29(96.7)	1(3.3)	29(96.7)	1(3.3)	30(100)	0
−1	0		1(3.3)		0	
Unchanged	26(86.7)		28(93.3)		30(100)	
+1	2(6.7)		0		0	
+3	1(3.3)		0		0	
Restored after visit		2(6.7)		0		0
**Day 180**	23(76.7)	7(23.3)/2(6.7)	28(93.3)	2(6.7)/0	28(93.3)	2(6.7)/0
−1	0		0		0	
Unchanged	19(63.3)		28(93.3)		28(93.3)	
+1	4(13.3)		0		0	
Restored after visit		0		0		1(3.3)
**Day 360**	23(76.7)	7(23.3)/2(6.7)	27(90)	3(10)/0	27(90)	3(10)/1(3.3)
−1	0		2(6.7)		6 (20)	
unchanged	16(53.3)		25(83.3)		21(70)	
+1	7(23.3)		0		0	
Restored after visit		4(13.3)		0		0
Total restored during trial	6(20.0)		0		1(3.3)	
	**Control vs Test 1**		**Control vs Test 2**		**Test 1 vs Test 2**	
**Odds’ Ratio (95 CI%)**^**a**^	1.20 (1.05–1.37)		1.27 (1.10–1.47)		1.05 (0.96–1.15)	
**p-value**	**0.008**		**0.001**		**0.260**	

^a^Tested effect: interaction of months and treatment group of GEE (general. Estimation Equation) adjusted for: ICDAS-II at Day 0, sex, age, plaque index at Day 0.

^b^Missing data due to restorations at a previous visit.

### Nyvad Activity Criteria

All three study groups had a comparable distribution of active and inactive caries lesions at baseline (Table 1). Changes in the Nyvad Activity Criteria throughout the study are described in Table 4. In the Control Group, the inactive/active lesions changed from 4/26 at baseline to 13/10 at Day 360. Test Group 1 showed superior caries inactivation to the Control Group, as the inactive/active lesions increased from 1/29 (Day 0) 27/0 (Day 360) (p < 0.0005). Test Group 2 showed superior caries inactivation to the Control Group, as the inactive/active lesions increased from 3/27 (Day 0) to 27/0 (Day 360) (p = 0.002). There were no statistical significant differences between the two Test Groups (p = 0.205).

**Table 4:** Nyvad Activity Criteria and Changes in Control and Test Groups at baseline and 90, 180, and 360 days of follow-up.

	Control Group	Test Group 1	Test Group 2	Test vs Control^a^
**Nyvad Caries Activity Criteria**				
**D0**				
Active	26	29	27	
Inactive	4	1	3	
**D90**				
Active	24	4	5	Test 1: p** < **0.0005
Inactive	5	25	25	Test 2: p** < **0.0005
**D180**				
Active	15	0	1	Test 1: p** < **0.0005
Inactive	8	28	27	Test 2: p** < **0.0005
**D360**				
Active	10	0	0	Test 1: p = 0.001
Inactive	13	27	27	Test 2: p < 0.0005
		**Control vs Test 1**	**Control vs Test 2**	**Test 1 vs Test 2**
**Odds’ Ratio (95 CI%)**^**a**^		4.60 (2.17–9.90)	3.00 (1.49–6.00)	0.56 (0.23–1.38)
**p-value**^**b**^		**<0.005**	**0.002**	0.205

^a^Binary logistic regressions, corrected for: Nyvad at Day 0, sex, age, plaque index Day 0.

^b^Tested effect: interaction of months and treatment group of GEE (general. Estimation Equation) adjusted for: Nyvad at Day 0, sex, age, plaque index Day 0.

### Visual Analogue Scale for surface texture

**Figure 1:**
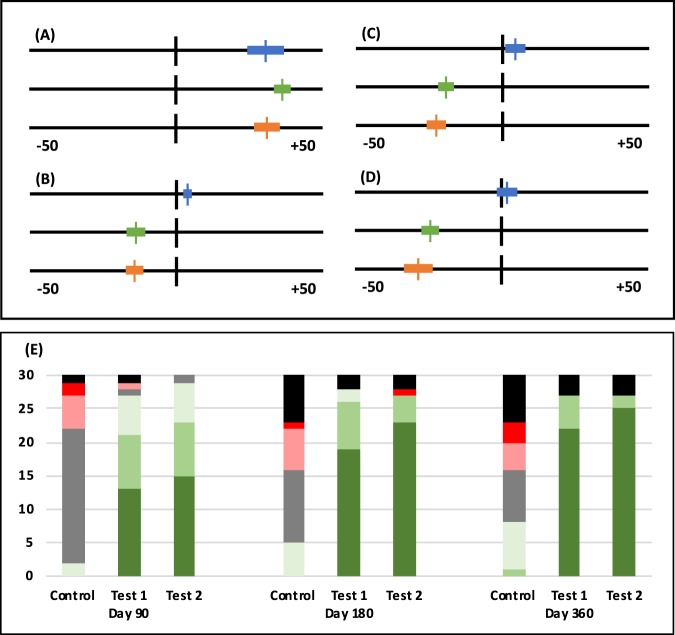
Investigator Assessments of the study patients. (**A–D**) Visual Analogue Scale for Tactile Assessment. The lines indicate the 10-cm VAS scale ranging from −50 to +50. Negative values indicate a harder and smoother surface, and positive values indicate softer and rougher surface. Top Control Group (blue), middle Test Group 1 (green), bottom Test Group 2 (orange). The mean value is indicated by a vertical line, and the bars indicate the 95% Confidence Interval. (**A**) Baseline; (**B**) Day 90; (C) Day 180; (**D**) Day 360. (**E**) Bar chart representing the number of answers to the Global Impression of Change questionnaire for Control, Test Group 1 and Test Group 2 on the indicated follow-ups for each answer. The answers are colour coded: Worse (red); A little worse (light red); Unchanged (grey); A little better (light green); Better (green); Very much better (dark green); Missing answers (black).

All three study groups showed comparable Visual Analogue Scale (VAS) values for surface texture at baseline (Table 1). The VAS values are shown in Fig. 1. VAS values for surface texture of the lesion (tactile assessment) of the Control Group showed overall stabilization on all three follow-up visits. Test Group 1 and Test Group 2 showed continuous hardening/smoothing of the enamel that was superior to the Control Group according to the VAS scores throughout the follow-up visits (p < 0.0005). There was no statistical difference observed between Test Groups 1 & 2 (p = 0.647).

### Global Impression of Change questionnaire

The Global Impression of Change (GOIC) questionnaires at the three follow-up visits showed an overall worsening of the caries in the Control Group and a clear superior improvement of the carious enamel of Test Groups 1 & 2 (p < 0.0005) (Fig. 1). There was no statistical difference observed between Test Groups 1 & 2 (p > 0.05).

### Adverse events

No adverse events or safety issues were observed throughout the study in any study group.

## Discussion

In the present study, both Test Groups, including SAP P_11_-4 either with fluoride varnish or with SAPM, showed superior results in terms of enamel remineralisation compared to the Control Group treated with fluoride varnish. As would be expected, the Control and Test Group 1 show similar results as in the previous clinical study with a similar design including those two treatment groups^16,22,23^. In addition, it also showed similar results as other randomized controlled trials investigating SAP P_11_-4 or SAPM.

Differences to Alkilzy *et al*. 2017 could be seen in the magnitude of the laser fluorescence signal decrease for both Test Groups compared to the previous study^16^, yet this might have been due to the overall lower starting values in the present study. The relative decreases of laser fluorescence among the SAP P_11_-4 groups in the studies were similar in the two studies. The corroborating ICDAS-II code data were also similar, showing between 6.7–20% regression into lower ICDAS-II codes in the present study and similar numbers in the previous study^16^. The laser fluorescence readings of the Control Group in the present study increased slightly (non-significant) and remained unchanged in the previous study^16^.

The present trial addressed critical questions raised by the previous trials on SAP P11-4. As there was no statistically significant difference between the two Test Groups, it suggests that the effect of Fluoride Vanish 2x per year leads to similar results as SAPM 2x per week, if used in combination with SAP P_11_-4, and thus SAPM could be substituted for fluoride varnish treatment without loosing effectiveness. Secondly, erupted molars and premolars benefit similarly from the treatment as did erupting molars. Finally the regenerative process supported by SAP P_11_-4 mostly occurs within the first 3 months as can be seen by the inactivation of the caries and the laser fluorescence values, but regression of the carious enamel could be observed throughout the year, especially changes in ICDAS-II codes.

The underlying rationally designed biomimetic technology of SAP P_11_-4 has gone through rational development and clinical testing. It was first investigated within the basic sciences^25^, then entered oral biology^11^ and afterwards translated into clinical practice in a combined effort of academia and industry^16,18,19,21,23^. In recent years, several clinical studies, both in university clinic and private practice settings, have been reported, all supporting the effectiveness and safety of SAP P_11_-4 and its superiority over other conventional remineralizing agents, such as Fluoride^16,18,19,21,23^.

To adapt our clinical practice and use SAP P_11_-4 as a routine prevention measure, the clinical relevance of the superiority seen in SAP P_11_-4 over fluoride varnish should be discussed. The main advantage shown in the clinical trial – and in this one in particular – is that SAP P_11_-4 can initiate a regression for the caries and not just an arresting/inactivation of the lesions, as observed in many fluoride studies. To preserve the natural tooth structure as long as possible and delay a restorative intervention, a regression of the caries, by a biomimetic agent such as SAP P_11_-4, is a significant clinical benefit. For the first time, such a guided enamel regeneration enables the tooth to get closer to its healthy state and further from the disease state^3,26^. Arresting a lesion with fluoride stops the imminent clinical issue at hand, but does little to alleviate the thread of a lesion becoming active again and progressing to a cavity^27^.

Based on the clinical benefit provided by, and the bulk of scientific and clinical data available for SAP P_11_-4 in the treatment of carious lesions, a translation into every day practice is imminent and should be welcomed by the profession as a new effective tool to decrease caries rates.

The present clinical study is not without limitations. The main limitation is the blinding within the study, which was limited to Control Group and Test Group 1 patients. This might have affected the Nyvad Activity Criteria and ICDAS-II assessments and could have led to a bias for the subjective assessments, VAS Scores, and GOIC. However, as these assessments corroborate the primary parameter of laser fluorescence, obtained with a validated device, there seems to be robustness for the data as a whole.

Scientifically, it would have been interesting to provide SAPM (Test Group 2) without the 900-ppm fluoride. However, as fluoride toothpaste was used throughout the study, the investigators felt that the additional 900 ppm fluoride twice a week supplied by SAPM had little effect on the overall outcome of the clinical trial.

## Conclusion

The data obtained from the clinical trial showed SAP P_11_-4, applied either in combination with fluoride varnish or twice weekly SAPM, to be a superior treatment for early caries lesions compared to fluoride varnish alone. The efficacy of the two groups including SAP P_11_-4 did not show differences in the treatment of occlusal caries as in the present study no statistically significant differences were observed. These findings are based on laser fluorescence, ICDAS-II Code, Nyvad Caries Activity Criteria and investigator assessments.

## Materials and Methods

### Study design

The present study was designed as a randomized, gold-standard controlled, single blinded clinical trial comparing the combined treatment of a single application of SAP P_11_-4 and fluoride varnish (Test Group 1) and the combined treatment of a single application of SAP P_11_-4 and a twice weekly self-application of SAPM (Test Group 2) against the gold standard fluoride varnish applied twice per year (Control).

All assessments were performed in accordance with ISO 14155:2011 Clinical investigation of medical devices for human subjects - Good clinical practice and the national regulation for clinical trials in the Republic of Kosovo. Subjects were recruited from the daily clinical practice of the paediatric dental department of the University Dentistry Clinical Center of Kosovo and fulfilled the following selection criteria:

#### Inclusion criteria

- Present early occlusal carious lesions (ICDAS-II codes 2 or 3) that do not require invasive treatment
- Age ≥ 6 years and ≤ 15 years
- Size and form of the carious lesions must both be fully visible, assessable, and accessible
- Willing and able to attend the on-study visits and to observe good oral hygiene throughout the study
- Provide written informed consent before participation in the study

As subjects were minors, informed consent was provided by the parent and/or legal guardian.

#### Exclusion criteria

- Evidence of tooth erosion
- Fluoride varnish application <3 months prior to study treatment
- History of head and neck illnesses (e.g., head/neck cancer)
- Any pathology or concomitant medication affecting salivary flow or dry mouth
- Any metabolic disorders affecting bone turnover
- Concurrent participation in another clinical trial

To ensure similar fluoride levels among subjects, all subjects were asked whether they use fluoride tablets or drops in addition to their fluoride toothpaste, which all subjects declined. There is no water-, milk-, or salt-fluoridation anywhere in Kosovo. If a subject presented more than one early carious lesion, then all lesions where treated according to the study allocation, but only one lesion was included in the assessment. Within the study, lesions on molars surpassed lesions on pre-molars.

### Randomization and blinding

Subjects were randomly assigned to either of the 3 study groups by drawing consecutively numbered envelopes providing the study group assignment. The randomization envelopes were provided by a third party. As a single investigator performed the treatment and all assessments, blinding of the investigator was not possible. Patients were only blinded with regards to Test Group 1 and Control Group, as Test Group 2 patients were given home applications of SAPM.

### Treatment

Subjects were treated according to their study group allocation: Control Group lesions were treated with fluoride varnish (Fluor Protector S (7′700ppm Fluoride, Ivoclar Vivadent, Schaan, Lichtenstein) at baseline and Day 180; Test Group 1 lesions were treated once with SAP P_11_-4 at baseline (Curodont Repair, credentis, Windisch, Switzerland), including pretreatment according to the instructions for use: Fluoride-free prophypaste (Proxypaste RDA 36 fluoride free, Ivoclar Vivadent, Schaan, Lichtenstein), 2% NaOCl (Chloraxid, Cerkamed, Poland), and 35% Etch Gel (Voco, Cuxhaven, Germany), followed by fluoride varnish application. Test Group 1 received fluoride varnish application at Day 180 as the control group did. Test Group 2 lesions were treated once with SAP P_11_-4 at baseline identical to Test Group 1 but without fluoride varnish application. Instead, SAPM (Curodont Protect, credentis, Windisch, Switzerland) was provided, and study patients were instructed on the application. SAPM was to be applied twice per week with a finger. Subjects of Test Group 2 were provided with a calendar to monitor SAPM application.

All study materials used in the present study, especially SAP P_11_-4 and SAPM are suitable and recommended by the manufacturer for the use in children and adolescents.

Study patients were asked to attend follow-up visits at Day 90, Day 180 and Day 360; Control Group and Test Group 1 received an additional fluoride varnish application at Day 180.

### Study visits and assessments

Baseline assessments were performed after written informed consent for study participation was provided by all study patients prior to study group assignment (i.e., randomization) and first study treatments.

All assessments at all study visits were performed by a single trained investigator as no alternative clinical investigator trained in both GCP and study procedures was available at the study site. The following assessments were performed only at baseline: Simplified Oral Hygiene Index (OHI-S)^28^, Plaque Index, Caries Risk Assessment^29^, DMFT. The following assessments were performed at each study visit: Laser Fluorescence (DiagnoDent Pen, KaVo, Biberach, Germany) - Triple measurements were performed, and the average was taken for statistical analysis; ICDAS-II code of the study lesion (not including activity of the carious lesion)^4^; Nyvad Activity Criteria assessment of the study lesion^30^; Visual Analogue Scale (VAS) for Lesion Texture Assessment (tactile) on a scale of −50 to +50, where negative values indicate hard(er)/smooth(er) lesions, “0” unchanged lesions, and positive values soft(er)/rough(er) carious lesions^21,31^; Global Impression of Change Questionnaire (GIOC) was assessed by the investigator at all follow-up visits^21^. The GIOC is a seven-point scale with answers ranging from: “very much worse”, “worse”, “a little worse”, “unchanged”, “a little better”, “better”, and “very much better”.

### Statistical analysis

A power analysis was performed on the bases of the interim results of the clinical trial of Alkilzy *et al*. 2018.

To explore if the development of the examined parameters during the 1-year study period was different between the treatment groups, tests of interaction of time and group were performed in the framework of multilevel models.

For metric parameters - laser fluorescence, VAS texture assessment – these multilevel models were linear mixed models, while ICDAS-II class, Nyvad Activity Status and GOIC were analysed in the framework of generalized estimation equation (GEE) for ordinal outcome variables; all were adjusted for their corresponding baseline values, sex, age, and plaque index.

In addition to these global tests, group differences in progression in the tested parameters and changes from baseline to day 90, day 180 and day 360 were assessed. Pairwise comparisons between the treatment groups with respect to the changes from baseline to day 90, day 180 and day 360 were performed by means of ANCOVA or ordinal logistic regressions, depending on the scale of the outcome variables. Again, the models were adjusted for their corresponding baseline values, sex, age, and plaque index.

### Knowledge transfer statement

The results of this study should aid clinicians in their decision on whether non-invasive early caries treatment might be suitable for a patient. In addition, it educates the clinician on how to quantify the success of a non-invasive treatment and what changes to expect. This can lead to a more prevention-orientated treatment decision and thus less invasive dentistry, enabling the patient to keep their natural teeth longer.

## Supplementary information

Supplementary information

Supplementary information2

## Data Availability

The datasets generated during and analysed during the current study are available from the corresponding author on request.
