# Supplementary material for: Randomized Clinical Trial investigating Self-Assembling Peptide P11-4 for Treatment of Early Occlusal Caries
Source: Sci Rep. 2020 Mar 6;10:4195. doi: 10.1038/s41598-020-60815-8 (PMC7060217; doi:10.1038/s41598-020-60815-8)

Randomized Clinical Trial investigating Self-Assembling Peptide P_{11}-4 for Treatment of Early Occlusal Caries

Authors: Dafina Doberdoli[1,2], Claudine Bommer[3], Agim Begzati[1], Fehim Haliti[1], Monika Heinzel-Gutenbrunner[4], Hrvoje Juric[2]

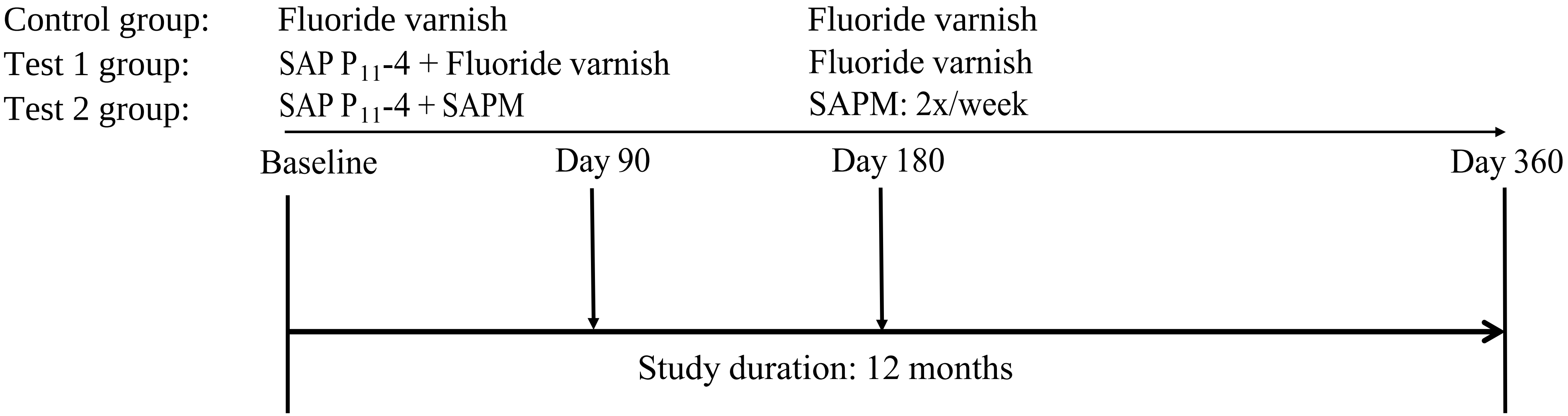

Supplement: Supplementary file 2 — Supplementary information2 [file 41598_2020_60815_MOESM2_ESM.pdf]
